# Matrix-Bound Nanovesicles as Tissue-Specific Signaling Hubs for Immunomodulation and Precision Regenerative Medicine

**DOI:** 10.3390/pharmaceutics18070857

**Published:** 2026-07-14

**Authors:** Peyton M. Leyendecker, George S. Hussey

**Affiliations:** 1 McGowan Institute for Regenerative Medicine, Pittsburgh, PA 15219, USA; pel150@pitt.edu; 2 Department of Bioengineering, University of Pittsburgh, Pittsburgh, PA 15261, USA; 3 Department of Pathology, University of Pittsburgh, Pittsburgh, PA 15213, USA; 4 Department of Surgery, University of Pittsburgh, Pittsburgh, PA 15213, USA

**Keywords:** matrix-bound nanovesicles (MBVs), extracellular matrix (ECM), extracellular vesicles (EVs), regenerative medicine, tissue-specific bioactivity, macrophage polarization, interleukin-33 (IL-33), surface engineering, cargo loading, precision medicine

## Abstract

The evolution of regenerative medicine has repositioned the extracellular matrix (ECM) from a passive structural scaffold to a dynamic signaling hub that dictates host immunity and tissue remodeling. A critical driver of this bioactivity is the matrix-bound nanovesicle (MBV), a distinct subclass of extracellular vesicles (EVs) physically embedded within collagen fibers. Unlike fluid-phase EVs, MBVs exhibit unique release kinetics triggered by matrix degradation and possess tissue-specific molecular signatures that dictate their therapeutic potential. This review evaluates the biogenesis, isolation, and cellular tropism of MBVs, highlighting the macrophage as a central mediator of their immunomodulatory effects. We propose a “precision medicine” framework for matching MBV tissue sources—ranging from pro-angiogenic small intestinal submucosa to anti-angiogenic cartilage—to the specific pathological requirements of the target injury. Furthermore, we discuss post-harvest engineering strategies, including surface functionalization via click chemistry and exogenous cargo loading, to enhance MBV targeting and potency. Finally, we address the translational hurdles of protocol standardization and pharmacokinetic characterization required to transition MBVs into a scalable, cell-free platform for regenerative therapy.

## 1. Introduction

The extracellular matrix (ECM) has undergone significant reconceptualization in regenerative medicine over the past decade. Once regarded as a passive structural scaffold providing mechanical support to tissues, the ECM is now recognized as a dynamic signaling hub that mediates cellular behavior, immune responses, and tissue remodeling [1,2]. This shift has been driven in part by the discovery that ECM-based biomaterials retain bioactive components capable of influencing host cell phenotypes and functions beyond the capacity of structural proteins alone [3,4].

A critical advance in this understanding is the identification of matrix-bound nanovesicles (MBVs), a distinct subclass of extracellular vesicles (EVs) physically embedded within the collagen fibers of tissues [4]. First described by Huleihel et al. in 2016, MBVs are characterized as nanosized, lipid membrane-bound vesicles residing within the ECM that carry microRNA (miRNA) and protein cargo capable of influencing cell behavior [4]. While MBVs share morphological similarities with exosomes, they differ fundamentally in their anatomical localization and mechanism of release [5]. Unlike conventional exosomes or microvesicles found in bodily fluids, MBVs are bound to the ECM fibers and are released upon enzymatic degradation of the matrix—a process that occurs naturally during tissue injury and remodeling, or artificially during the preparation of ECM-based scaffolds [4]. This “on-demand” release mechanism positions MBVs as important mediators of the host immune response, delivering bioactive cargo when and where the tissue is undergoing active remodeling [3].

The broader EV field has experienced explosive growth, with EVs emerging as novel tools for diagnostics, drug delivery, and immunotherapy [6]. These lipid bilayer-enclosed bodies are secreted by virtually all cell types and carry bioactive materials like proteins, lipids, and nucleic acids that mediate intercellular communication and induce functional changes in recipient cells [7]. The therapeutic potential of EVs is underscored by their high biocompatibility, biological barrier permeability, and low immunogenicity [8]. However, clinical translation of EV-based therapeutics has been limited by challenges including limited yields, rapid phagocytic clearance, and non-specific biodistribution after injection [9]. MBVs, by virtue of their unique origin within the solid-state matrix, may offer distinct advantages in terms of cargo stability, tissue-specific bioactivity, and controlled release kinetics that could address some of these translational hurdles [4,5,10].

In the context of the pharmaceutical sciences, understanding the full potential of MBVs requires a systematic examination of their tissue-specific signatures, cellular uptake mechanisms, and capacity to be engineered as modular platforms for targeted drug delivery. This review examines the expanding MBV literature while leveraging established EV frameworks to build evidence-informed foundations for future MBV-based therapeutics.

## 2. Biogenesis, Composition, and Isolation of MBVs

### 2.1. MBV Biogenesis and ECM Integration

MBVs represent a unique category within the EV landscape [4,5]. While the biogenesis of conventional exosomes involves the endosomal pathway, where multivesicular bodies fuse with the plasma membrane to release vesicles, MBVs are thought to be deposited directly into the ECM during its synthesis and remodeling by resident cells [3]. Once embedded, MBVs become integral components of the matrix architecture, which protects them from degradation and clearance mechanisms that typically eliminate circulating, fluid-phase EVs [4]. This integration within the ECM gives MBVs a degree of stability and longevity that is unmatched by their fluid-phase counterparts [4,11].

The molecular composition of MBVs reflects both their cellular origin and their tissue microenvironment [12,13,14]. Like other EVs, MBVs contain proteins, lipids, and nucleic acids that can modulate recipient cell behavior [3,4]. However, the specific proteomic and miRNA profiles of MBVs are dictated by their tissue of origin, creating a “tissue-specific signature” that varies across different anatomical sites [12,14].

### 2.2. Isolation Methods and Their Impact on MBV Properties

The isolation of MBVs from decellularized tissues requires enzymatic digestion of the ECM to liberate the embedded vesicles [5,15], a process fundamentally distinct from the isolation of circulating EVs. While fluid-phase EVs are typically recovered via ultracentrifugation or size-exclusion chromatography from biological fluids [15], MBV recovery depends heavily on the specific enzyme and digestion methods used. These conditions directly dictate the resulting yield, purity, and functional integrity of the isolated vesicles [6].

Systematic evaluations of various solubilization approaches, including collagenase, liberase, proteinase K, and nonenzymatic elution, demonstrate that while most combinations successfully harvest MBVs, their biological signatures vary significantly [15]. For instance, pairing liberase or collagenase digestion with size-exclusion chromatography (SEC)-based isolation consistently produces the highest yields and superior cellular uptake [15]. Conversely, harsher treatments, such as proteinase K digestion combined with ultrafiltration, have demonstrated detrimental effects on vesicle bioactivity [15]. This sensitivity to processing conditions emphasizes the need for optimized, standardized isolation protocols to ensure consistent performance and effective clinical translation.

Across the MBV literature, a variety of enzymatic approaches have been employed for various parent tissues, reflecting the diversity of isolation methods. Table 1 summarizes the enzymatic and separation strategies employed to recover MBVs from various tissue types.

The methodological heterogeneity across different tissue sources and the effect of these methods on MBV bioactivity reflect a broader challenge within the EV field regarding protocol standardization [7,15]. While the Minimal Information for Studies of Extracellular Vesicles (MISEV) guidelines provide a rigorous framework for conventional EVs [7], analogous criteria for MBVs remain absent [15]. The indiscriminate use of different methods can compromise the structural and functional integrity of MBVs, making method selection a critical variable for therapeutic reproducibility and clinical translation [15].

The choice of enzymatic agent influences MBV properties not only through digestion efficiency but also through the molecular specificity of the enzyme itself. Collagenase and liberase formulations act preferentially on the collagen scaffold that anchors MBVs, liberating vesicles while leaving their surface proteome comparatively intact [5,15]. Broad-spectrum proteases such as proteinase K, by contrast, cleave a wide range of peptide bonds and can degrade the surface ligands that promote cellular recognition [21]. This mechanistic distinction provides a likely explanation for the functional differences observed across protocols. Vesicles released under milder, collagen-selective conditions likely retain the membrane features required for efficient internalization, whereas more aggressive proteolysis can strip these determinants and diminish bioactivity even when nanoparticle yield is preserved [15]. Enzyme concentration, incubation time, and temperature represent additional variables that further modulate the balance between adequate matrix solubilization and preservation of vesicle integrity.

Isolation does not end with the liberation of the vesicles from the matrix. The subsequent separation and purification step is a second determinant of preparation quality. Once MBVs have been released from the digested ECM, they must be separated from residual matrix fragments, free proteins, apoptotic bodies, and the digestion enzymes themselves. Ultracentrifugation remains the most widely used approach but can co-pellet smaller matrix debris and protein aggregates with the MBVs; SEC generally yields higher purity samples but with lower yield; ultrafiltration, while convenient and scalable, has been associated with reduced bioactivity, particularly when paired with harsher digestion [15]. Because the liberation chemistry and the separation method interact, the same parent tissue can yield MBVs with markedly different purity, yield, and potency depending on the specific combination employed, complicating direct comparison across studies.

Finally, the properties of the final MBV product are shaped not only by isolation but by the characterization and handling steps that follow. Consistent reporting of particle concentration and size distribution by nanoparticle tracking analysis (NTA), morphology by transmission electron microscopy (TEM), and molecular cargo (miRNA, protein, and lipid content) is necessary to determine whether vesicles recovered by different protocols are in fact comparable [7,15]. Pre-analytical factors such as freeze-thaw cycling and storage temperature, which are known to affect the stability and function of conventional EVs [7,22,23], are likely to influence MBVs as well but remain largely uncharacterized. Establishing standardized characterization and criteria, analogous to the MISEV framework for conventional EVs [7] but adapted to the MBV, it will therefore be as important as optimizing the isolation itself for ensuring reproducible MBV preparations.

### 2.3. Distinguishing MBVs from Other Extracellular Vesicles

**Figure 1:**
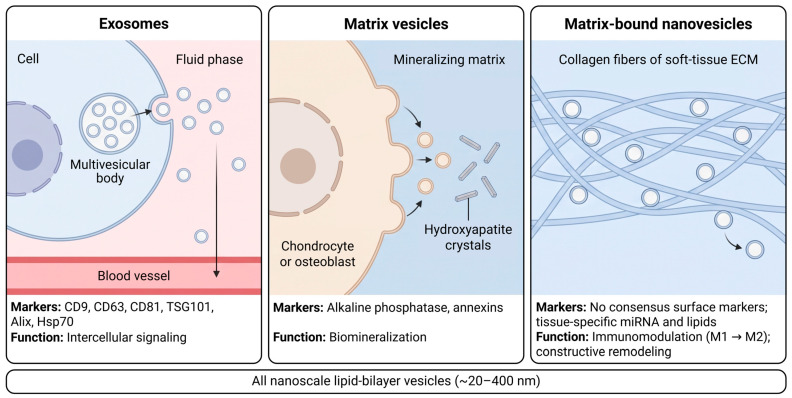
Distinguishing matrix-bound nanovesicles (MBVs) from other extracellular vesicles. Exosomes arise from the endosomal/multivesicular body pathway and are released into the fluid phase, carrying tetraspanins (CD9, CD63, CD81) and biogenesis-associated markers. Matrix vesicles bud from chondrocytes and osteoblasts to nucleate hydroxyapatite in the mineralizing matrix and are defined by alkaline phosphatase and annexins. MBVs are bound within the collagen of soft-tissue extracellular matrix (ECM), lack a consensus surface marker signature, and carry tissue-specific miRNA and lipid cargo associated with immunomodulation and constructive remodeling. All are nanoscale lipid-bilayer vesicles (~20–400 nm).

Although the MBVs characterized to date are frequently grouped with EVs because of their nanoscale size and lipid bilayer, molecular characterization indicates that this resemblance is largely superficial (Figure 1) [4,5]. Using liquid chromatography, mass spectrometry, lipidomics, and RNA sequencing, Hussey et al. showed that the similarity between MBVs and liquid-phase EVs is largely limited to the size and shape [5]. MBVs possess a distinct phospholipid speciation, a distinct protein cargo, and a unique miRNA signature relative to exosomes secreted by the same cells [5]. This distinction is particularly evident at the level of surface markers. Exosomes are defined by enrichment in the cluster of differentiation (CD) endosomal tetraspanins CD9, CD63, and CD81, together with proteins such as tumor susceptibility gene 101 (TSG101), ALG-2 interacting protein X (Alix), and heat shock protein 70 (Hsp70) [7], whereas MBVs lack or express only markedly reduced levels of these canonical markers. In the original characterization of MBVs, CD63, CD81, CD9, and Hsp70 were undetectable in bioscaffold-derived MBVs by immunoblot [4]. Another EV that emphasizes the novelty of MBVs is the matrix vesicle of mineralizing tissues (also known as biomineralization vesicles). Like MBVs, matrix vesicles are physically associated with the ECM rather than the fluid phase, but they are produced by chondrocytes, osteoblasts, and odontoblasts, are defined by tissue-nonspecific alkaline phosphatase and annexins, and serve to nucleate hydroxyapatite during bone, cartilage, and dentin mineralization [4,24]. MBVs are therefore distinguished from exosomes by their solid-phase localization and divergent marker profile [4,5], and from matrix vesicles by their soft-tissue origin and immunomodulatory rather than mineralizing function [4,24]. These differences reinforce the view that MBVs constitute a distinct vesicle population whose tissue-specific signatures guide their therapeutic behavior [5]. Other extracellular vesicle subclasses, including microvesicles and apoptotic bodies, share the fluid-phase, non-collagen-bound character of exosomes and are likewise distinguished from MBVs. For a comprehensive comparison of MBVs with the major extracellular vesicle classes, see the recent review by Di Francesco et al. [25].

## 3. MBV Uptake and Internalization

### 3.1. General Principles of EV Uptake

The therapeutic efficacy of any EV-based therapeutic, including MBVs, is fundamentally dependent on its ability to be internalized by specific recipient cells, a phenomenon known as cellular tropism [11,26,27]. This internalization is a highly regulated process governed by vesicle surface composition, recipient cell membrane receptors, and the immediate microenvironmental context [28,29]. Multiple pathways facilitate this entry, including clathrin-mediated and caveolae-dependent endocytosis, macropinocytosis, phagocytosis, and direct membrane fusion [28]. Because native EVs inherit cell adhesion molecules and ligands from their parent cell membranes [30], they possess innate targeting specificities that dictate their biodistribution and therapeutic impact [27,31].

### 3.2. MBV-Specific Uptake Considerations

Unlike conventional fluid-phase EVs, MBVs originate within the solid-state matrix, resulting in a unique surface proteome [4]. For example, MBVs derived from brain organoids contain a higher density of membrane proteins, such as integrins, compared to supernatant-derived EVs [32]. These proteins likely contribute to MBV retention within the matrix and significantly influence their interaction with recipient cells.

Beyond innate proteomic differences, the enzymatic digestion required to liberate these vesicles from the ECM introduces a critical variable. Rather than just affecting yield, these digestion conditions can alter the surface properties, shifting the preferred internalization pathways or target cell populations [15]. This is evidenced by the fact that liberase/SEC-isolated MBVs exhibit significantly higher internalization rates compared to those recovered via harsher enzymatic treatments, suggesting that preservation of the surface landscape is necessary for efficient cellular entry [15].

Physical dimensions further complicate these dynamics. MBVs typically exhibit size heterogeneity ranging from 20 to 400 nm [13], a size range that encompasses both exosome-like and microvesicle-like particles. While MBVs often cluster in the 100 to 150 nm range [32], there can be broader size distributions depending on tissue source and isolation method [14,15]. This diversity in size likely triggers a variety of uptake mechanisms, as smaller vesicles tend to be internalized via clathrin-mediated endocytosis, while larger vesicles may be taken up via macropinocytosis or phagocytosis [33].

### 3.3. Macrophages

The most extensively characterized target cell for MBVs is the macrophage [3]. As seen in the broader EV field, vesicles are predominantly sequestered by macrophages and other phagocytic cells of the mononuclear phagocyte system [34]. While rapid clearance often limits the bioavailability of fluid-phase EVs for non-phagocytic targets, this natural tropism provides a strategic advantage for therapeutic platforms designed to drive macrophage reprogramming [3,16].

Labeled MBVs are internalized by bone marrow-derived macrophages (BMDMs) within two hours of exposure, establishing a profile of rapid and efficient uptake [3]. However, as established in Section 2.2, this internalization is highly sensitive to isolation methods. While liberase/SEC-isolated vesicles show high uptake, proteinase K treatment significantly reduces this interaction [15]. This suggests that specific surface ligands essential for macrophage recognition are susceptible to proteolytic degradation during processing. The downstream biological consequences of macrophage internalization of MBVs—including phenotypic polarization, transcriptomic reprogramming, and disease-modifying effects—are discussed in detail within the tissue-specific therapeutic applications in Section 5.

### 3.4. Neurons, Microglia, and Astrocytes

Evidence indicates that MBVs interact with both neuronal and glial populations. Direct endocytosis has been observed in hippocampal neurons, where fluorescein-labeled UBM-ECM MBVs localize within filopodia, growth cones, neurites, and cell bodies as early as 15 min after exposure [35]. These rapid kinetics likely reflect the active endocytic machinery of neuronal growth cones and the high surface-to-volume ratio of neuritic projections. Furthermore, the capacity for neurons to internalize and respond to these signals appears independent of the tissue source or decellularization method. For instance, MBVs extracted from brain and placenta tissues decellularized via high-hydrostatic pressure (HHP) have both been shown to promote neurite outgrowth in vitro [36].

Beyond direct neuronal interaction, MBVs modulate the behavior of the surrounding glial cells [37]. In the context of neuroinflammation, MBVs suppress pro-inflammatory signaling in activated astrocytes and microglia, effectively shifting the glial environment toward a neuroprotective state [37]. This modulation has functional consequences for retinal health, as MBV treatment protects retinal ganglion cells (RGCs) from the neurotoxic media typically produced by pro-inflammatory astrocytes [37]. While the precise uptake mechanisms for glia remain to be characterized at the single-pathway level, their functional responses confirm they are active recipients of MBV signaling. As the resident macrophages of the central nervous system (CNS), microglia likely internalize MBVs through phagocytic mechanisms similar to those of peripheral myeloid cells, whereas astrocyte uptake may involve distinct endocytic pathways that warrant further exploration. The downstream neuroregenerative and neuroprotective effects of MBV-neural cell interactions are discussed in Section 5.1.6 and Section 5.5.

### 3.5. Epithelial Cells

Both normal (KTB21) and cancerous (MDA-MB-231) mammary epithelial cells can internalize MBVs, whether from isolated preparations or directly from decellularized tissue sections [19]. Notably, uptake rates are significantly higher in cancer cells, a phenomenon potentially linked to the increased metabolic flux characteristic of pathological populations [19]. This differential uptake suggests that cancer cells may be more susceptible to MBV-mediated signaling than their normal counterparts. Furthermore, MBVs can be embedded in collagen gels to mimic the natural ECM context, where they are subsequently released and taken up by surrounding cells. The biological consequences of MBV uptake by epithelial and cancer cells are discussed in Section 5.7 and Section 5.8.

### 3.6. Stem Cells and Fibroblasts

MBV signaling extends to both stem cells and stromal populations. Vesicles isolated from 3D mesenchymal stem cell (MSC) cultures are internalized by forebrain organoids and high-passage fibroblasts [38]. Similarly, MBVs derived from vocal fold lamina propria exhibit functional uptake by fibroblasts, as evidenced by subsequent modulation of gene expression [18]. While these cell types are clearly responsive to MBV signaling, the specific endocytic pathways and kinetics involved have not yet been characterized with the same level of detail as myeloid or neural targets. The therapeutic implications of MBV-fibroblast interactions are discussed in Section 5.4 and Section 5.6.

### 3.7. Osteoclasts and Bone Cells

In the skeletal system, MBVs have been shown to attenuate receptor activator of nuclear factor kappa-B ligand (RANKL)-induced osteoclast differentiation and activity [17]. Although the specific endocytic pathways mediating this interaction are not yet defined, osteoclasts are derived from the monocyte-macrophage lineage and likely share the phagocytic mechanisms of their myeloid precursors. The capacity to modulate osteoclast behavior expands the known cellular tropism of MBVs into orthopedic and bone-healing applications, which are discussed in Section 5.1.3.

### 3.8. Vascular Endothelial Cells

Vascular endothelial cells are also direct recipients of MBV signaling. Successful internalization has been demonstrated using human umbilical vein endothelial cells (HUVECs), which show significant intracellular accumulation of labeled MBVs within two hours of exposure [12]. Additionally, MBVs induce functional changes in the endothelial cell population related to proliferation and angiogenesis [12,13], indicating successful internalization and cargo delivery. Insights from the broader EV field suggest that this endothelial tropism could be further enhanced by targeting surface-conjugated peptides, such as RGD-based ligands, to reactive vascular beds [39]. The specific influence of the tissue source on endothelial response is a key variable in determining angiogenic potential. These therapeutic applications are discussed in Section 5.2 and Section 5.11.

### 3.9. T Cells and Adaptive Immune Cells

While direct uptake studies of MBVs by T cells are limited, systemic MBV administration has profound effects on T cell populations in vivo. In an influenza model, MBV increased the proportion of activated anti-viral CD4+ and CD8+ T cells at day 7 and memory-like CD62L+ CD44+ T cells at day 21 [10]. In a rheumatoid arthritis model, MBV treatment was associated with the emergence of a systemic CD43hi/His48lo/CD206+ immunoregulatory monocyte population [16]. Whether these T cell effects result from direct MBV-T cell interactions or are secondary to MBV-mediated macrophage reprogramming remains an important open question. But, evidence suggests that the genes regulated by MBV-associated interleukin-33 (IL-33) in macrophages are involved in innate-adaptive crosstalk [40], suggesting that MBV-mediated macrophage reprogramming may indirectly shape adaptive immune responses through paracrine signaling and antigen presentation.

### 3.10. Dendritic Cells

As professional antigen-presenting cells, dendritic cells (DCs) are central to initiating adaptive immunity. Although direct evidence for MBV-DC interactions is limited, the broader literature demonstrates that EVs modulate DC maturation and antigen presentation [41]. In murine models of volumetric muscle loss, UBM-derived ECM has been observed to induce a response from multiple myeloid cell populations, including dendritic cells [42]. This suggests that DCs are responsive to ECM-derived signals, likely including the embedded MBVs found within these scaffolds.

If MBVs carry similar surface ligands to those found on tissue-resident EVs or can be engineered to target DCs, they could serve as potent modulators of adaptive immunity. Matrix sources with specialized immune environments, such as the liver, may yield MBVs with inherent DC-targeting properties, as discussed in Section 5.9.

### 3.11. Fibroblast-like Synoviocytes

Fibroblast-like synoviocytes (FLS) are primary drivers of inflammation and progression in rheumatoid arthritis [43]. Given that MBVs reduce synovial inflammation in arthritis models [16], it is plausible that they interact with FLS either directly or through macrophage-mediated paracrine signaling. Like MSC-derived exosomes, which inhibit FLS migration and proliferation [44,45], MBVs may offer a localized means of suppressing the inflammatory synovial phenotype. The therapeutic implications of potential MBV-FLS interactions in the context of rheumatoid arthritis are discussed in Section 5.1.1.

### 3.12. Summary

The breadth of cell types demonstrated or proposed to internalize MBVs underscores the versatility of MBVs as intercellular signaling agents. Table 2 summarizes the current evidence for MBV uptake across cell types, distinguishing between directly demonstrated uptake and inferred or speculative interactions.

## 4. The Macrophage as a Central Mediator of MBV Bioactivity

The capacity to reprogram macrophages from a pro-inflammatory (M1-like) to a pro-remodeling (M2-like) phenotype is the most consistently demonstrated and therapeutically significant property of MBVs [3,40,47,48]. This property appears to be a conserved feature that transcends the tissue of origin, although the specific molecular mediators and the magnitude of the effect can vary with tissue-specific cargo [3,47]. For example, MBVs isolated from both porcine urinary bladder and small intestinal submucosa recapitulate the macrophage activation effects of their parent bioscaffolds, sharing an enrichment of miR-125b-5p, miR-143-3p, and miR-145-5p [3,48]. While overall polarization is conserved, tissue-specific differences can modulate the character of the response, such as the increased phagocytic activity observed with UBM-derived vesicles compared to those from SIS [3]. The presence of IL-33 across UBM, SIS, dermis, and cardiac-derived MBVs—as well as in commercially available products—further supports IL-33-mediated reprogramming as a conserved mechanism [4,40,47]. This immunomodulatory potential extends to cell culture-derived MBVs, which modulate polarization through various cytokine pathways, and remains effective regardless of the decellularization method employed [1,13,32,38].

The molecular mechanisms underlying this conserved property involve at least three complementary pathways. First, IL-33 within MBVs can bypass classical IL-33/suppression of tumorigenicity 2 (ST2) receptor signaling to direct macrophage differentiation via a non-canonical, ST2-independent mechanism [40,47]. RNA sequencing has identified over 2000 differentially expressed genes in macrophages exposed to these vesicles, primarily related to the inflammatory response and innate-adaptive immune crosstalk [40]. Second, specific miRNAs—including miR-125b-5p, miR-143-3p, and miR-145-5p—mediate anti-inflammatory effects across multiple tissue sources [3]. Third, MBVs are enriched in pro-resolving lipid precursors, such as arachidonic acid, docosahexaenoic acid, and docosapentaenoic acid, which can be metabolized into lipoxin A4 and resolvin D1 to stimulate M2-like activation [5,25,49].

An important nuance is that the resulting macrophage phenotype often deviates from the classical M2 paradigm. The phenotype elicited by ECM exposure (designated M_ECM_) is distinct from canonical M_IFNγ+LPS_ and M_IL-4_ phenotypes [3]. Observations of scaffold-associated macrophages expressing both CD206 (M2-like) and CD86 (M1-like) markers suggest the existence of intermediate subtypes between M1 and M2 [50]. Consequently, MBV-mediated reprogramming produces a nuanced, context-dependent phenotype that is broadly anti-inflammatory and pro-remodeling but not fully captured by simplified frameworks. Having established macrophage polarization as a conserved mechanism, the following section examines different MBV tissue sources and their unique bioactive signatures beyond macrophage modulation.

## 5. Tissue-Specific MBVs and Their Potential for Precision Medicine

A defining and clinically relevant feature of MBVs is their tissue-specific identity. Comprehensive examinations of MBVs isolated from diverse tissue sources demonstrate that while these vesicles are ubiquitous within the ECM, they contain protein and microRNA cargo that is unique to their tissue of origin [14]. This principle was further supported by distinct cytokine and protein profiles observed across different matrices. For example, MBVs from decellularized bovine pericardium exhibit unique signatures, such as the expression of interferon-alpha (IFN-α) and the absence of angiopoietin-1, which contrast with the profiles observed in other characterized MBV sources/tissues. [1,14].

Lipidomic and RNA sequencing analyses further reinforce this specificity, showing that MBVs isolated from ECM produced in vitro by bone marrow, adipose, and umbilical cord stromal cells each harbor distinctive miRNA signatures dictated by the cell source [5]. Characterizations of vesicles from anatomically distinct sources, including UBM, SIS, and dermis, similarly reveal differential miRNA signatures and distinct protein cargo profiles [4].

This tissue-specific variation in MBV composition has profound implications for therapeutic applications. For clinical translation, the “one-size-fits-all” approach to regenerative medicine should be replaced by a precision framework where the MBV source is matched to the specific pathological requirements of the injury or disease. The following subsections examine each characterized MBV source, its unique bioactive signature, how it modulates recipient cell behavior, and the disease contexts in which its therapeutic potential has been demonstrated or can be reasonably hypothesized.

This translational rationale for matching MBV source to indication is reinforced by the extensive clinical history of the decellularized ECM materials from which these vesicles derive. Decellularized ECM bioscaffolds are already in widespread clinical use, with hundreds of products on the market and several million patients treated [51,52], with the immunomodulatory, pro-remodeling activity of these materials now attributed in substantial part to their MBV content [25]. The application-specific evidence summarized below should therefore be read against this established ECM precedent: although direct clinical data for isolated MBVs are not yet available and the supporting studies remain preclinical, the clinical track record of the source biomaterials provides meaningful translational context for each tissue source.

### 5.1. UBM-Derived MBVs

Urinary bladder matrix (UBM)-derived MBVs have demonstrated potent effects across the broadest range of disease models to date. The anti-inflammatory signature likely reflects the unique immunological environment of the urinary bladder, which must maintain tolerance to urine-associated antigens while remaining responsive to infection. While the macrophage-polarizing properties of UBM-derived MBVs are shared with other tissue sources [48], UBM-derived MBVs have been uniquely validated in numerous in vivo contexts, establishing them as a benchmark for MBV-based therapies.

#### 5.1.1. Rheumatoid Arthritis

Both intravenous and peri-articular administration of UBM-derived MBVs have been shown to reduce arthritis scores in acute and chronic pristane-induced models. This treatment decreases synovial inflammation and adverse joint remodeling while successfully shifting the ratio of synovial and splenic macrophages from an M1 to an M2 phenotype [16]. Notably, the efficacy of MBVs is comparable to methotrexate, the current clinical standard, while also promoting a unique systemic CD43hi/His48lo/CD206+ immunoregulatory monocyte population [16].

These findings demonstrate that MBV-mediated immunomodulation operates at both local (synovial) and systemic (splenic) levels, evidenced by reduced serum concentrations of key inflammatory biomarkers like C-X-C motif chemokine ligand 10 (CXCL10) and monocyte chemotactic protein 3 (MCP-3) [16]. By shifting macrophages toward a pro-remodeling state, these UBM MBVs interrupt the inflammatory cascade between fibroblast-like synoviocytes and T cells that typically drives joint destruction [16]. Furthermore, MBVs offer a simplified alternative to fluid-phase exosome therapies, requiring significantly less processing and manipulation [16].

#### 5.1.2. Influenza and Viral-Mediated Cytokine Storm

In models of influenza-mediated acute respiratory distress syndrome, intravenous MBV administration significantly decreases total lung inflammatory cell density and pro-inflammatory cytokine levels [10]. These effects persist through both the acute and recovery phases, reducing long-lasting alveolitis and the extent of pathological tissue repair [10].

Importantly, MBV treatment increases the proportion of activated anti-viral CD4+ and CD8+ T cells and memory-like T cell populations. This suggests that the therapy does not compromise the anti-viral immune response but rather enhances it while simultaneously dampening pathological inflammation [10]. While UBM-derived MBVs are effective, the potential for further improvement using lung-specific vesicles remains a compelling argument for a precision medicine framework [10].

#### 5.1.3. Periprosthetic Osteolysis

Local administration of UBM-derived MBVs has been shown to attenuate osteolysis induced by ultrahigh molecular weight polyethylene particles, promoting bone reconstruction and reducing periosteal inflammation [17]. Mechanistically, these vesicles attenuate RANKL-induced osteoclast differentiation by suppressing the NF-κB (nuclear factor kappa-light-chain-enhancer of activated B cells) signaling pathway and downstream expression of markers like NFATc1 (nuclear factor of activated T-cells, cytoplasmic 1), DC-STAMP (dendritic cell-specific transmembrane protein), and cathepsin K [17]. Consistent with established mechanisms, MBV treatment also promotes the polarization of periosteal macrophages toward a pro-remodeling M2 phenotype [17].

#### 5.1.4. Skeletal Muscle Injury

The therapeutic potential of UBM-derived MBVs in muscle repair is largely driven by their IL-33 cargo. This cytokine supports skeletal muscle regeneration by regulating local macrophage activation via both canonical and non-canonical pathways [40,47,53]. Research utilizing IL-33 knockout models indicates that host responses and functional recovery following skeletal muscle injury are profoundly impaired in the absence of this signaling, whereas the administration of IL-33-containing MBVs significantly restores functional outcomes [53].

#### 5.1.5. Corneal Wound Healing

The application of UBM-derived particulate to corneal wounds promotes type 2 immune responses, substantially reducing the corneal haze formation compared to saline controls [54]. This treatment up-regulates interleukin-4 (IL-4) production, primarily mediated by eosinophils in the wounded tissue and CD4+ T cells in the draining lymph nodes, suggesting a crosstalk between local and peripheral immunity [54]. While this study used UBM particulate rather than isolated MBVs, the natural degradation of UBM releases MBVs, indicating that MBVs alone may also be effective for the observed tissue repair.

#### 5.1.6. Neurological and Neuro-Ophthalmic Applications

UBM-derived MBVs have demonstrated remarkable neuroprotective and neuroregenerative properties. In hippocampal contexts, they have been shown to increase neuron survival and stimulate neurite growth [35]. Notably, evidence also suggests that non-homologous tissue sources like UBM may provide an effective, economical, and safer alternative to CNS-derived matrices for these applications [35]. Finally, UBM-derived MBVs have been shown to regulate differentiation and axon growth in neuroblastoma and primary CNS neurons [55].

In the context of severe intraocular pressure-induced ischemia, intravitreal MBV injections attenuate RGC axon degeneration, preserve axon connectivity to the brain, and prevent the loss of retinal function [37]. MBVs also prevent the decrease in growth-associated protein-43 (GAP-43) while dampening the increase in GFAP, a marker of reactive gliosis [37]. In vitro studies confirm that MBVs suppress pro-inflammatory signaling in activated microglia and astrocytes, protecting RGCs from neurotoxic media.

More recently, combination strategies have further enhanced these effects. Pairing UBM-derived MBVs with fluvastatin robustly promotes the infiltration of immunomodulatory monocytes and neutrophils, leading to enhanced RGC protection and axon regeneration after optic nerve crush injury [46].

The breadth of demonstrated applications for UBM-derived MBVs—ranging from autoimmune arthritis to viral cytokine storm to periprosthetic osteolysis to skeletal muscle regeneration to corneal wound healing to ischemic retinal neuroprotection to optic nerve regeneration—underscores their versatility and establishes them as the most clinically advanced MBV source to date.

Beyond this preclinical scope, the clinical behavior of the parent material aligns closely with the activity attributed to its vesicles: U.S. Food and Drug Administration (FDA)-cleared decellularized UBM devices promote a shift away from a chronic inflammatory environment toward M2-macrophage polarization, cellular infiltration, and tissue regeneration [56,57,58]. This clinically observed immunomodulation and constructive remodeling support the premise that UBM-derived MBVs recapitulate the bioactivity of the parent matrix.

### 5.2. SIS-Derived MBVs

Small intestinal submucosa (SIS) is arguably one of the most widely commercialized and clinically utilized ECM sources [59,60], with several FDA-approved devices currently in use for wound care and surgical repair [59,61]. Because of this established manufacturing infrastructure and the abundance of the parent tissue, SIS-derived MBVs may represent the most cost-effective source for large-scale clinical translation.

Historically, SIS-based materials have been recognized for their ability to shift the biochemical balance of chronic wounds toward an acute, healing state [62]. This transition is driven by the capacity of the matrix to influence macrophage polarization and stem cell differentiation [62]. Building on this, isolated SIS-derived MBVs (sMBVs) have demonstrated significant potency in promoting vascular endothelial cell proliferation [13,14]. Recent systematic characterizations confirm that sMBVs recapitulate the inherently pro-angiogenic properties of the parent SIS matrix [12]. These vesicles are enriched with a specific suite of pro-angiogenic miRNAs—including miR-143-3p, miR-181a, and miR-21-5p—which promote capillary network formation and endothelial cell migration [12].

Beyond vascular effects, SIS-derived MBVs carry a potent immunomodulatory signature. They are enriched with miRNAs such as miR-125b-5p and miR-145-5p, which promote M2-like macrophage activation [3], and they have been confirmed to carry IL-33 [47]. The functional impact of these signals is evident in models of ulcerative colitis, where SIS-based hydrogels delivered via enema result in a marked reduction in clinical and histologic disease signs, including the restoration of colonic epithelial barrier function and the mitigation of pro-inflammatory macrophage phenotypes [63,64].

The bioactivity of SIS-derived MBVs also extends to the nervous system. Vesicles extracted from HHP-decellularized SIS have been shown to promote neurite outgrowth, suggesting a broader regenerative capacity that includes neuroprotection [36]. While direct disease-model studies using isolated sMBVs are currently less numerous than those for UBM, their demonstrated ability to drive endothelial proliferation and resolve inflammation makes them ideal candidates for precision applications in diabetic wound healing, peripheral arterial disease, and post-ischemic tissue repair.

### 5.3. Bovine Pericardium-Derived MBVs

The isolation of MBVs from decellularized bovine pericardium has revealed a complex repertoire of proteins and cytokines relevant to tissue repair and vascular remodeling [1]. These vesicles are composed of various bioactive factors, including acidic and basic fibroblast growth factors (FGFs), the anti-inflammatory mediator insulin-like growth factor-1 (IGF-1), and the immunomodulatory proteoglycan decorin [1]. Furthermore, evidence of IL-33 within cardiac ECM-derived matrices suggests that pericardium-derived MBVs likely carry this critical immunomodulatory cargo [47]. Given the cardiovascular origin of the parent tissue, these MBVs may possess signals specifically tailored to cardiac repair [1]. While bovine pericardium has long been utilized in cardiovascular surgery, the discovery that its decellularized ECM harbors bioactive MBVs adds a significant new dimension to the biological understanding of these established clinical materials [1].

In considering translational potential, it is relevant that decellularized bovine pericardium is a long-established clinical biomaterial used in bioprosthetic heart valves and as pericardial, vascular, and dural patches [65]. Notably, decellularized pericardium promotes host-cell repopulation, neovascularization, and appropriate remodeling of cardiovascular tissue [65,66]. This capacity for constructive remodeling provides translational context for pericardium-derived MBVs.

### 5.4. Vocal Fold Lamina Propria-Derived MBVs

MBVs isolated from vocal fold lamina propria (VFLP) exhibit a distinct antifibrotic signature, characterized by the capacity to downregulate *ACTA2* expression even under transforming growth factor beta 1 (TGF-β1) stimulation [18]. This tissue-specific behavior suggests that VFLP-derived MBVs carry specialized signals that counteract fibrotic responses, positioning them as prime candidates for vocal fold repair [18]. Because the VFLP possesses a unique ECM composition optimized for vibration and phonation, its embedded MBVs may recapitulate these properties. Whether the antifibrotic cargo could be applied to fibrotic conditions in other organs remains an intriguing but untested hypothesis.

### 5.5. Brain and Placenta-Derived MBVs

The identification of MBVs within brain organoids and placental matrices has expanded the possibilities for neural-specific regenerative medicine. In models of ischemic stroke, brain organoid-derived MBVs demonstrate superior recovery effects compared to supernatant-derived EVs, primarily through the regulation of autophagy, scavenging of reactive oxygen species, and anti-inflammatory activity [32]. These vesicles are notably enriched in glycerophospholipids and sphingolipids that influence membrane rigidity, and their production within organoid cultures can be up to ten-fold higher than their fluid-phase counterparts [32]. Furthermore, the extraction of MBVs from tissues decellularized via HHP demonstrates that neuroregenerative vesicles can be recovered from both brain and placental sources to promote neurite outgrowth and nerve fiber repair [36].

The identification of placental MBVs is particularly significant for clinical translation, as the placenta represents an abundant and ethically accessible tissue source [67]. Beyond neural applications, the placenta’s role in maternal-fetal tolerance suggests its MBVs may harbor unique tolerogenic signatures applicable to autoimmune disease or transplant tolerance [68]. While UBM-derived MBVs have shown robust efficacy in acute neuroinflammatory conditions, tissue-matched cargo from brain-derived MBVs may offer specialized advantages in chronic neurodegenerative states [35,36].

The placental side of this source also has a substantial clinical history that reinforces this rationale: amniotic (placental) membrane is a long-established graft in ophthalmology and wound care, where its clinical benefit is largely attributed to anti-inflammatory, anti-scarring, and pro-epithelialization activity [69,70,71]. This immunomodulatory mode of action parallels that attributed to MBVs.

### 5.6. 3D MSC Culture-Derived MBVs

The culture microenvironment profoundly influences MBV properties, with three-dimensional (3D) MSC-derived vesicles exhibiting distinct immunomodulatory profiles and enhanced expression of miRNAs, such as miR-19a and miR-21, compared to 2D counterparts [38]. While both 2D and 3D-derived MBVs modulate macrophage polarization, they appear to utilize different cytokine signaling pathways [38]. Functionally, 3D-derived MBVs facilitate recovery in forebrain organoids following starvation and stimulate the proliferation of high-passage fibroblasts [38]. These findings suggest that in vitro culture conditions can be engineered to produce MBVs with tailored properties, offering a potential scalable manufacturing approach. Furthermore, proteomic analysis confirms that MBVs derived from MSC cultures possess higher angiogenic potential than their liquid-phase counterparts [72].

### 5.7. Breast ECM-Derived MBVs

Research into breast ECM-derived MBVs reveals that their cargo is not static but evolves with the physiological state of the tissue. Specifically, the cargo profile shifts toward a more tumorigenic and invasive signature with age; MBVs isolated from aged tissues exhibit higher expression of “oncomiRs” such as miR-10b, miR-30e, and miR-210 [19]. When exposed to these aged MBVs, both normal and cancerous mammary epithelial cells demonstrate significantly increased motility and invasive capacity [19]. This demonstrates that MBV cargo is not static but changes with the physiological state of the tissue, with implications for understanding age-related disease susceptibility and potential biomarker development.

### 5.8. Tumor ECM-Derived MBVs

MBVs isolated from decellularized tumor ECM demonstrate a specialized capacity to target both parent tumor cells and tumor-associated stromal populations [20]. This suggests that MBVs carry tissue-of-origin “address labels” that facilitate homing to the specific source microenvironment [20]. While the conserved M2-promoting activity of most MBV sources might appear counterproductive in a tumor, the reality is more complex. For example, UBM-derived matrices containing IL-33+ MBVs have been shown to synergize with checkpoint blockade to inhibit tumor formation [40].

### 5.9. Liver-Derived MBVs

While functional characterization of liver-derived MBVs is in its early stages, their successful extraction from both standard and HHP-decellularized liver tissue has been confirmed [13,14]. Given the liver’s role in systemic immune tolerance—mediated by specialized populations like Kupffer cells and tolerogenic dendritic cells [73]—MBVs derived from this matrix likely harbor unique tolerogenic signatures [14].

### 5.10. Skin-Derived MBVs

Porcine skin represents a well-characterized source for MBV isolation [4]. Dermis-derived MBVs have been confirmed to carry IL-33, indicating that the conserved macrophage-polarizing mechanism extends to the skin [47]. Furthermore, these vesicles contain a highly diverse cargo profile, with up to 46 distinct miRNAs identified [4]. While direct disease-model studies are limited, potential applications include chronic wound management, burn treatment, and barrier restoration.

Among ECM-derived therapies, acellular dermal matrices are among the most widely adopted in clinical practice (burn care, chronic wound management, breast reconstruction, and hernia repair) [74]. Beyond providing structural support, they encourage angiogenesis, host-cell repopulation, and constructive tissue integration [74,75,76]. The clinical performance of dermal ECM materials provides translational context for dermis-derived MBVs; their IL-33-bearing, miRNA-rich cargo offers a plausible basis for that activity [4].

### 5.11. Cartilage-Derived MBVs

Perhaps the most intriguing tissue-specific MBV signature is that of cartilage-derived MBVs. Articular cartilage maintains stability by resisting vascular invasion through endogenous anti-angiogenic factors. Recent evidence confirms that MBVs isolated from this matrix (cMBVs) recapitulate these properties by selectively packaging anti-angiogenic cargo [12]. Unlike SIS-derived vesicles, cMBVs demonstrate a potent capacity to inhibit endothelial cell proliferation and migration, driven by a distinct profile of miRNAs—most notably miR-140-3p and miR-455-5p [12].

#### 5.11.1. Wet Age-Related Macular Degeneration

The anti-angiogenic potency of cartilage-derived MBVs suggests a different therapeutic approach for wet age-related macular degeneration (AMD) [77,78]. Evidence from corneal neovascularization models shows that cMBV treatment significantly reduces vascular area [12]. This builds upon the established precedent for ocular MBV administration, where UBM-derived MBVs have been used to preserve visual function after injury [37,46].

#### 5.11.2. Osteoarthritis and Joint Repair

Cartilage-derived MBVs also hold promise for the treatment of osteoarthritis, where they could help restore the homeostatic environment of the joint [79]. Because cMBVs carry endogenous, cartilage-specific signals, they may represent a more physiologically relevant approach to joint repair than exogenously loaded EVs [12].

The parent material itself has a clinical foundation: micronized decellularized cartilage allograft matrices are used clinically to augment microfracture repair of chondral and osteochondral lesions, where they act not simply as a scaffold but as a chondroinductive material that supplies bioactive signals supporting chondrogenesis [80,81,82]. This signaling role of the parent matrix supports a cartilage-repair application for cartilage-derived MBVs, contingent on the isolated vesicles recapitulating the chondroinductive activity of their source matrix, as MBVs from other tissues have been shown to do.

### 5.12. Matching MBV Source to Therapeutic Need

**Figure 2:**
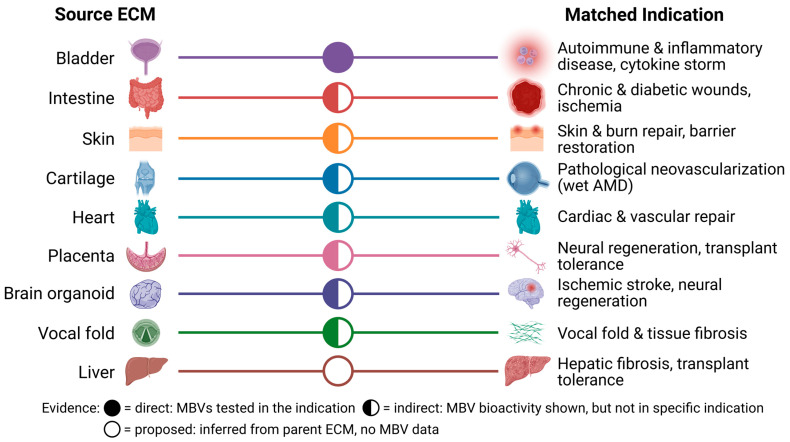
Matching matrix-bound nanovesicle (MBV) source to therapeutic indication. Each source extracellular matrix (ECM) is linked to the indication(s) suggested by its bioactive signature. Badges grade how directly the isolated-MBV evidence supports that specific match: filled = direct (isolated MBVs tested in the indication); half = indirect (isolated MBVs show the relevant bioactivity, but not in the specific indication); open = proposed (inferred from the parent ECM, no direct isolated-MBV data). Many of the parent ECMs are clinically established, whereas evidence for isolated MBVs remains largely preclinical. AMD = age-related macular degeneration.

The tissue-specific nature of MBVs creates an opportunity for precision medicine approaches in which the MBV source is selected based on the specific immunomodulatory and biological requirements of the target condition. As established in Section 4, all characterized MBV sources share the conserved macrophage-polarizing property and the tissue-specific cargo adds specialized bioactivity tailored to particular therapeutic contexts. Figure 2 and Table 3 summarize the proposed matching framework, linking each MBV source to its matched indication and the maturity of the supporting evidence.

This precision framework represents a significant departure from conventional EV-based therapeutics, which typically rely on cell culture-derived vesicles without tissue-specific context. The framework also highlights the substantial gap between the well-characterized UBM-derived MBVs and the many other tissue sources that remain largely speculative, underscoring the need for systematic comparative studies across MBV sources in standardized disease models.

## 6. Post-Harvest Enrichment and Engineering of MBVs

**Figure 3:**
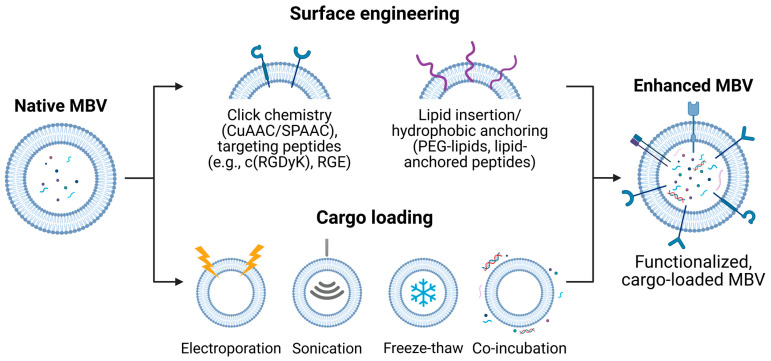
Engineering strategies for matrix-bound nanovesicles (MBVs). Native MBVs can be modified by surface engineering to add targeting and stabilizing functionalities, and by cargo loading to introduce therapeutic molecules, yielding a functionalized, cargo-loaded “enhanced MBV.” These approaches are adapted from the broader extracellular-vesicle engineering field and have not yet been validated on MBVs specifically. Within each vesicle, internal symbols represent cargo: the native MBV contains its endogenous, source-derived cargo (tissue-specific miRNA, lipids, and protein), while the enhanced MBV additionally carries exogenous therapeutic cargo (e.g., miRNA, siRNA, or small-molecule drugs) plus surface projections representing targeting ligands. Icons are illustrative and not drawn to scale.

While native MBVs are inherently powerful immunomodulatory agents, their transition into the pharmaceutical “toolbox” requires strategies to enhance their potency, targeting specificity, and reproducibility. The broader EV engineering field provides an extensive repertoire of modification strategies, including surface engineering and cargo loading, that can be adapted for MBVs (Figure 3).

### 6.1. Surface Engineering Strategies

The cellular tropism of EVs, as characterized in Section 3, is governed by surface ligands that are likely sensitive to isolation and processing conditions [15]. Modifying these vesicles to target specific cell types represents a potential strategy for expanding clinical applications [17]. For instance, encapsulating vesicles with specific cell membrane fragments [17,83,84] or adding synthetic antibodies and peptides can create a “forced” tropism [85,86,87], ensuring the vesicles home to desired cell populations with higher efficiency than their native counterparts.

#### 6.1.1. Click Chemistry

Click chemistry, specifically copper-catalyzed azide-alkyne cycloaddition (CuAAC) and strain-promoted alkyne-azide cycloaddition (SPAAC), has emerged as one of the most widely used methods for vesicle surface functionalization [88,89,90,91]. These reactions allow for the attachment of stable targeting ligands, such as antibodies or peptides, without altering the fundamental physical characteristics of the vesicle [88,90]. Validation studies on conventional EVs show that such modifications do not compromise vesicle size, adhesion, or internalization by recipient cells [90], suggesting that similar approaches could be applied to MBVs without compromising their native bioactivity.

Specific applications of this technology include the conjugation of the c(RGDyK) peptide to target integrin αvβ3 in reactive/activated vascular endothelial cells [92] and the conjugation of neuropilin-1-targeted peptide (RGE) for glioma targeting [93]. Such strategies are particularly relevant for the tissue-specific applications discussed in Section 5. For example, SIS-derived MBVs could be functionalized with vascular-targeting peptides to amplify their pro-angiogenic effects in ischemic tissues. Conversely, for ocular applications such as wet AMD, cartilage-derived MBVs could be conjugated with ligands targeting the retinal pigment epithelium (RPE) or choroidal vasculature to enhance the local delivery of anti-angiogenic cargo. Because MBVs already possess inherent targeting affinity to their tissue of origin [20], click chemistry could further enhance this natural homing capability.

#### 6.1.2. Lipid Insertion and Hydrophobic Anchoring

Non-covalent modification through hydrophobic insertion offers a simpler alternative to covalent chemistry [94]. This approach involves inserting lipophilic or amphiphilic molecules—such as poly(ethylene glycol) (PEG)-modified lipids or lipid-anchored peptides—directly into the MBV lipid bilayer. This technique can be used to introduce PEG molecules (PEGylation) to extend circulation time or to display multiple targeting moieties simultaneously [95,96]. This method is particularly advantageous for displaying complex receptors and co-receptors on the MBV membrane.

### 6.2. Cargo Loading Strategies

Beyond surface modifications, the loading of exogenous therapeutics into MBVs represents another avenue for pharmaceutical enrichment. Multiple cargo loading strategies have been developed for conventional EVs, including both passive and active approaches [97,98,99,100,101]. These strategies could allow for the creation of “enriched MBVs” that combine their natural bioactivity with targeted, standardized pharmaceutics.

#### 6.2.1. Electroporation

Electroporation utilizes brief electrical pulses to temporarily permeabilize the vesicle membrane, facilitating the insertion of siRNAs, miRNAs, or small-molecule drugs [102,103,104]. This technique could be used to load MBVs with therapeutic agents not natively present in the matrix. For example, UBM-derived MBVs could be loaded with additional anti-inflammatory miRNAs to enhance their efficacy in treating severe cytokine storms beyond the baseline effects provided by their natural cargo [10].

#### 6.2.2. Sonication

Sonication momentarily disrupts the vesicle membrane structure, allowing co-incubated substances to diffuse into the vesicle interior during membrane reassembly [104,105]. Sonication has been shown to achieve higher loading efficiency than co-incubation or electroporation for certain cargo types [106,107]. However, sonication may alter the size of the vesicles and alter the protein and RNA content [105,107,108], which could affect MBV bioactivity. Careful optimization of sonication parameters would be necessary to balance cargo loading efficiency with preservation of native MBV function.

#### 6.2.3. Freeze-Thaw Cycles

The freeze-thaw method involves repeated cycles of freezing and thawing to temporarily disrupt the vesicle membrane, allowing cargo incorporation [99,100]. This remains one of the most accessible and popular methods for modifying vesicles for targeted drug delivery due to its simplicity and the relative lack of specialized equipment required [101].

#### 6.2.4. Co-Incubation

Passive co-incubation relies on concentration gradients and hydrophobic interactions to drive cargo incorporation into the lipid bilayer [101,109]. Although it typically results in lower loading efficiency than active methods [110], this gentle approach is more likely to preserve the native surface ligands critical for cellular tropism. For MBVs, co-incubation could be used for the loading of hydrophobic drugs that can incorporate into the lipid bilayer.

MBVs hold great promise as a platform for the delivery of targeted gene and drug therapeutics [111]. The “enriched MBV” concept would combine the low immunogenicity and natural cloaking of a biological vesicle with the potency of a standardized drug. Proof-of-concept studies have already demonstrated the success of loading MBVs with chemotherapeutic agents like doxorubicin, showing that the resulting platform can successfully target both primary tumor cells and their associated stromal environment [20].

## 7. Challenges for Clinical Translation

Despite the compelling preclinical evidence for MBV-based therapeutics, several significant challenges regarding standardization, manufacturing, and biological characterization must be addressed before these vesicles can transition into clinical practice.

### 7.1. Standardization of Isolation and Characterization

The most pressing challenge remains the lack of standardized protocols for MBV isolation. Current research utilizes a diverse array of enzymatic approaches—ranging from various Liberase isoforms (TL [16], DL [5], and TH [20]) to specific Collagenase types (I, II [18,19], and IV)—which complicates cross-study comparisons. As established in Section 2.2, these isolation methods yield vesicles with vastly different profiles in terms of purity, yield, and bioactivity [15]. Harsher protocols, such as those combining proteinase K with ultrafiltration, can be detrimental to functional potency, whereas milder enzymatic digestion paired with size-exclusion chromatography (SEC) appears to better preserve vesicle integrity [15].

To achieve clinical reproducibility, the field requires consensus guidelines analogous to the MISEV criteria established for conventional EVs [7]. These standards must define key parameters, including:

1. **Enzyme Specificity:** Standardized concentrations and digestion durations tailored to specific matrices
2. **Quality Control Metrics:** Mandatory reporting of size distribution (NTA), morphology (TEM), and detailed miRNA/lipid composition.
3. **Functional Benchmarks:** Validated bioactivity assays, with macrophage polarization serving as a baseline for immunomodulatory potential.
4. **Source Documentation:** Tracking of donor species, age, and anatomical site to account for inherent cargo variation.

### 7.2. Dose-Response and Pharmacokinetics

Rigorous dose-response relationships and pharmacokinetic profiles remain largely undefined for most MBV applications. Some dosing strategies have relied on in vitro macrophage activation thresholds [53], but these do not always translate linearly to complex in vivo environments. Establishing effective therapeutic windows—utilizing dose ranges such as 10^7^ to 10^11^ vesicles—is essential for ensuring efficacy while minimizing potential off-target effects.

Furthermore, the biodistribution of MBVs likely shifts in the presence of disease. While baseline models provide a foundation [11], future studies must clarify how inflammation or tissue injury alters the distribution and retention of MBVs across different administration routes, including intravenous, intravitreal, and topical applications. Systematic pharmokinetic studies in long-term survival models are required to guide clinical dosing regimens and determine the necessity for repeat administrations.

### 7.3. Tissue Source Selection

The precision medicine framework creates a strategic challenge in determining the optimal tissue source for specific clinical indications. While UBM-derived MBVs currently possess the broadest preclinical validation [10,16,17,25,35,37,46,53], evidence suggests that tissue-matched sources—such as lung-derived MBVs for respiratory distress [10] or brain-derived MBVs for chronic neurodegeneration—may offer superior efficacy. The absence of systematic, head-to-head comparisons in standardized disease models makes it difficult to determine when a “universal” immunomodulator is sufficient and when a specialized, tissue-matched cargo is required.

### 7.4. Functional Heterogeneity

MBV cargo is not a fixed signature. It reflects the physiological state of the parent tissue. Factors such as donor age and health status can profoundly shift the miRNA and protein profile of the isolated vesicles. For example, vesicles derived from aged matrices can exhibit shifts toward pro-inflammatory or even tumorigenic signatures [19], potentially introducing unwanted functional heterogeneity into the final product. Controlling for this biological variation necessitates rigorous quality control measures and a deeper understanding of how the physiological state of the source tissue dictates the therapeutic profile of the harvested MBVs.

## 8. Conclusions

MBVs have emerged as a distinct class of tissue-specific, immunomodulatory signaling entities with demonstrated efficacy across a diverse array of disease models. The current literature highlights several foundational themes that define this field.

First, MBVs possess a conserved macrophage-polarizing property that operates across virtually all characterized tissue sources. This universal immunomodulatory effect is mediated through the delivery of IL-33, specific miRNA signatures, and pro-resolving lipid mediators, which collectively drive a transition from pro-inflammatory to pro-remodeling phenotypes. Second, beyond these shared traits, MBVs harbor tissue-specific cargo that dictates specialized bioactivity. This is exemplified by the distinct vascular effects of these vesicles—ranging from the pro-angiogenic signatures of SIS-derived MBVs to the anti-angiogenic potential of cartilage-derived matrices—as well as the neuroprotective and antifibrotic properties of CNS and vocal fold-derived sources.

Third, the breadth of cell types capable of internalizing MBVs positions them as versatile intercellular signaling agents. This cellular tropism facilitates the delivery of bioactive cargo directly into the cytoplasm, bypassing classical extracellular ligand-receptor interactions. Fourth, the field has successfully transitioned from in vitro characterization to functional in vivo outcomes. Demonstrated successes include efficacy comparable to clinical standards in rheumatoid arthritis, the preservation of retinal ganglion cells following ischemic retinal injury, and enhanced nerve regeneration through combinatorial therapeutic strategies.

The precision medicine framework proposed in this review—matching the MBV tissue source to the specific therapeutic requirement—represents a conceptual advance that could transform the approach to both ECM-based and vesicle-based therapies. However, as outlined in Section 7, significant challenges remain regarding protocol standardization and the optimization of dose-response relationships. Ultimately, MBVs offer a unique platform for delivering the regenerative potential of the extracellular matrix. Realizing this potential will depend on the field’s ability to translate compelling preclinical evidence into standardized, scalable, and clinically validated therapeutic products.

## Figures and Tables

**Table 1:** Summary of matrix-bound nanovesicle (MBV) isolation methods utilized for different tissue types.

Tissue Source	Enzymatic Digestion Agent	Primary Separation Method	Key Reference(s)
Porcine Urinary Bladder (UBM)	Liberase (DL/TL) or Collagenase	Ultracentrifugation/Size-Exclusion Chromatography	[4,5,10,16,17]
Submucosal Small Intestine (SIS)	Collagenase	Ultracentrifugation	[12]
Bovine Pericardium	Liberase	Ultracentrifugation	[1]
Vocal Fold Lamina Propria	Collagenase II/Proteinase K	Ultrafiltration	[18]
Breast ECM	Collagenase II	Ultracentrifugation	[19]
Tumor-Derived ECM	Liberase TH	Ultracentrifugation	[20]

Abbreviations: ECM, extracellular matrix.

**Table 2:** Summary of evidence for matrix-bound nanovesicle (MBV) uptake and interaction across cell types.

Cell Type	Evidence Level	Key Uptake Findings	Reference(s)
Macrophages (BMDM)	Direct demonstration	Internalization within 2 h; uptake efficiency dependent on isolation method	[3,15,40]
Neurons	Direct demonstration	Endocytosis within ~15 min; vesicles observed in filopodia, growth cones, neurites, cell bodies; Neurite outgrowth promotion	[35,36]
Retinal Ganglion Cells	Functional evidence	MBV-mediated neuroprotection and axon preservation in vitro and in vivo	[37,46]
Microglia	Functional evidence	Suppression of pro-inflammatory signaling by activated microglia	[37]
Astrocytes	Functional Evidence	Suppression of pro-inflammatory signaling; GFAP modulation in vivo	[37]
Mammary Epithelial Cells	Direct demonstration	Uptake from isolated preparations and from decellularized tissue sections; higher uptake rate in cancerous epithelial cells than normal cells	[19]
Fibroblasts	Functional evidence	Modulation of *ACTA2* expression; promotion of proliferation	[18,32]
Stem Cells	Functional evidence	Facilitation of organoid recovery after starvation	[38]
Osteoclasts	Functional evidence	Attenuation of RANKL-induced differentiation; NF-κB suppression	[17]
Vascular Endothelial Cells	Direct demonstration	Intracellular accumulation of labeled MBVs within two hours; functional changes related to proliferation and angiogenesis	[12]
T Cells	Indirect/inferred	In vivo modulation of T cell populations; may be secondary to macrophage reprogramming	[10,40]
Dendritic Cells	Indirect/inferred	DC response to UBM-ECM in volumetric muscle loss model	[42]
Fibroblast-Like Synoviocytes	Speculative	Inferred from synovial inflammation reduction	[16]

Abbreviations: GFAP, Glial fibrillary acidic protein; RANKL, receptor activator of nuclear factor kappa-B ligand; NF-κB, nuclear factor kappa-light-chain-enhancer of activated B cells; DC, dendritic cell.

**Table 3:** Summary of tissue-specific matrix-bound nanovesicle (MBV) bioactivity, cellular targets, and proposed applications.

MBV Source	Key Bioactive Signatures	Primary Cell Targets	Proposed Therapeutic Application	Reference(s)
UBM	Anti-inflammatory (M1→M2 macrophage polarization); NF-κB suppression; neuroprotection	Macrophages, Osteoclasts, Neurons, RGCs, Microglia, Astrocytes, Monocytes, Neutrophils, FLS	Rheumatoid arthritis, viral cytokine storm, periprosthetic osteolysis, skeletal muscle injury, ischemic retinal injury/visual function preservation, CNS neuroprotection	[10,16,17,35,37,46,53]
SIS	Pro-angiogenic (endothelial cell proliferation); M2 macrophage activation, neurite outgrowth	Macrophages, Endothelial cells, Neurons	Ischemic conditions, chronic ulcers, diabetic wounds *, neural regeneration	[3,12,13,14,36,47]
VFLP	Antifibrotic (*ACTA2* downregulation)	Fibroblasts	Vocal fold fibrosis, other fibrotic conditions *	[18]
Brain (Organoid)	Neuroprotection; neurite outgrowth and nerve fiber repair	Neurons, Glia	Ischemic stroke, neural regeneration, neurological disorders *	[32,36]
Placenta	Neuroregenerative; tolerogenic potential *	Neurons, Schwann cells *	Neural regeneration, sciatic nerve repair, transplant tolerance *, autoimmune disease *	[36,67,68]
3D MSC Culture	Anti-inflammatory; pro-angiogenic	Macrophages, stem cells, fibroblasts	Neurological disorders, tissue regeneration *	[38]
Liver	Tolerogenic (immune regulation) *	Kupffer cells, DCs	Hepatic fibrosis *, transplant tolerance *, autoimmune hepatitis *	[14,73]
Cartilage	Anti-angiogenic	Endothelial cells, RPE *, Macrophages, Chondrocytes *	Wet AMD *, tumor angiogenesis *, pathological neovascularization *	[12,77,79]
Skin	Pro-regenerative (epithelial signals)	Epithelial cells, Fibroblasts	Chronic wounds, burns, barrier restoration *	[4,47]

* Indicates applications that are speculative based on tissue-specific cargo predictions rather than direct experimental evidence. Abbreviations: RGCs, retinal ganglion cells; FLS, fibroblast-like synoviocytes; RPE, retinal pigment epithelium.

## Data Availability

No new data were created or analyzed in this study. Data sharing is not applicable to this article.

## Abbreviations

The following abbreviations are used in this manuscript:

*ACTA2*	Alpha-smooth muscle actin
Alix	ALG-2 interacting protein
AMD	Age-related macular degeneration
BMDM	Bone marrow-derived macrophages
CD	Cluster of differentiation
CNS	Central nervous system
CuAAC	Copper-catalyzed azide-alkyne cycloaddition
CXCL10	C-X-C motif chemokine ligand 10
DC	Dendritic cell
DC-STAMP	Dendritic cell-specific transmembrane protein
ECM	Extracellular matrix
EV	Extracellular vesicle
FDA	U.S. Food and Drug Administration
FGF	Fibroblast growth factor
FLS	Fibroblast-like synoviocyte
GAP-43	Growth-associated protein-43
GFAP	Glial fibrillary acidic protein
HHP	High-hydrostatic pressure
Hsp70	Heat shock protein 70
HUVEC	Human umbilical vein endothelial cells
IFN-α	Interferon-alpha
IGF-1	Insulin-like growth factor-1
IL-33	Interleukin-33
LPS	Lipopolysaccharide
MBV	Matrix-bound nanovesicle
MCP-3	Monocyte chemotactic protein 3
MECM	ECM-educated macrophage phenotype
miRNA	microRNA
MISEV	Minimal Information for Studies of Extracellular Vesicles
MSC	Mesenchymal stem cell
NFATc1	Nuclear factor of activated T-cells, cytoplasmic 1
NF-κB	Nuclear factor kappa-light-chain-enhancer of activated B cells
NTA	Nanoparticle tracking analysis
PEG	Poly(ethylene glycol)
RANKL	Receptor activator of nuclear factor kappa-B ligand
RGC	Retinal ganglion cell
RPE	Retinal pigment epithelium
SEC	Size-exclusion chromatography
siRNA	Small interfering RNA
SIS	Small intestinal submucosa
SPAAC	Strain-promoted alkyne-azide cycloaddition
ST2	Suppression of tumorigenicity 2
TEM	Transmission electron microscopy
TGF-β1	Transforming growth factor beta 1
TSG101	Tumor susceptibility gene 101
UBM	Urinary bladder matrix
VFLP	Vocal fold lamina propria

## References

[1] Di Francesco D., Di Varsavia C., Casarella S., Donetti E., Manfredi M., Mantovani D., Boccafoschi F. (2024). Characterisation of Matrix-Bound Nanovesicles (MBVs) Isolated from Decellularised Bovine Pericardium: New Frontiers in Regenerative Medicine. Int. J. Mol. Sci..

[2] Yue B. (2014). Biology of the Extracellular Matrix: An Overview. J. Glaucoma.

[3] Huleihel L., Bartolacci J.G., Dziki J.L., Vorobyov T., Arnold B., Scarritt M.E., Pineda Molina C., LoPresti S.T., Brown B.N., Naranjo J.D. (2017). Matrix-Bound Nanovesicles Recapitulate Extracellular Matrix Effects on Macrophage Phenotype. Tissue Eng. Part A.

[4] Huleihel L., Hussey G.S., Naranjo J.D., Zhang L., Dziki J.L., Turner N.J., Stolz D.B., Badylak S.F. (2016). Matrix-Bound Nanovesicles within ECM Bioscaffolds. Sci. Adv..

[5] Hussey G.S., Pineda Molina C., Cramer M.C., Tyurina Y.Y., Tyurin V.A., Lee Y.C., El-Mossier S.O., Murdock M.H., Timashev P.S., Kagan V.E. (2020). Lipidomics and RNA Sequencing Reveal a Novel Subpopulation of Nanovesicle within Extracellular Matrix Biomaterials. Sci. Adv..

[6] Crum R.J., Capella-Monsonís H., Badylak S.F., Hussey G.S. (2022). Extracellular Vesicles for Regenerative Medicine Applications. Appl. Sci..

[7] Théry C., Witwer K.W., Aikawa E., Alcaraz M.J., Anderson J.D., Andriantsitohaina R., Antoniou A., Arab T., Archer F., Atkin-Smith G.K. (2018). Minimal Information for Studies of Extracellular Vesicles 2018 (MISEV2018): A Position Statement of the International Society for Extracellular Vesicles and Update of the MISEV2014 Guidelines. J. Extracell. Vesicles.

[8] Wiklander O.P.B., Nordin J.Z., O’Loughlin A., Gustafsson Y., Corso G., Mäger I., Vader P., Lee Y., Sork H., Seow Y. (2015). Extracellular Vesicle in Vivo Biodistribution Is Determined by Cell Source, Route of Administration and Targeting. J. Extracell. Vesicles.

[9] Luan X., Sansanaphongpricha K., Myers I., Chen H., Yuan H., Sun D. (2017). Engineering Exosomes as Refined Biological Nanoplatforms for Drug Delivery. Acta Pharmacol. Sin..

[10] Crum R.J., Huckestien B.R., Dwyer G., Mathews L., Nascari D.G., Hussey G.S., Turnquist H.R., Alcorn J.F., Badylak S.F. (2023). Mitigation of Influenza-Mediated Inflammation by Immunomodulatory Matrix-Bound Nanovesicles. Sci. Adv..

[11] Crum R.J., Capella-Monsonís H., Chang J., Dewey M.J., Kolich B.D., Hall K.T., El-Mossier S.O., Nascari D.G., Hussey G.S., Badylak S.F. (2023). Biocompatibility and Biodistribution of Matrix-Bound Nanovesicles in Vitro and in Vivo. Acta Biomater..

[12] Yun H.-W., Kim M., Shin D.I., Kwon H.J., Park I.-S., Park D.Y., Min B.-H. (2025). Matrix-Bound Nanovesicles Recapitulate Tissue-Specific Angiogenic Properties of Parent Extracellular Matrix with Distinct miRNA Profiles. Biomater. Adv..

[13] Kobayashi M., Ishida N., Hashimoto Y., Negishi J., Saga H., Sasaki Y., Akiyoshi K., Kimura T., Kishida A. (2022). Extraction and Biological Evaluation of Matrix-Bound Nanovesicles (MBVs) from High-Hydrostatic Pressure-Decellularized Tissues. Int. J. Mol. Sci..

[14] Turner N.J., Quijano L.M., Hussey G.S., Jiang P., Badylak S.F. (2022). Matrix Bound Nanovesicles Have Tissue-Specific Characteristics That Suggest a Regulatory Role. Tissue Eng. Part A.

[15] Quijano L.M., Naranjo J.D., El-Mossier S.O., Turner N.J., Pineda Molina C., Bartolacci J., Zhang L., White L., Li H., Badylak S.F. (2020). Matrix-Bound Nanovesicles: The Effects of Isolation Method upon Yield, Purity, and Function. Tissue Eng. Part C Methods.

[16] Crum R.J., Hall K., Molina C.P., Hussey G.S., Graham E., Li H., Badylak S.F. (2022). Immunomodulatory Matrix-Bound Nanovesicles Mitigate Acute and Chronic Pristane-Induced Rheumatoid Arthritis. npj Regen. Med..

[17] Liao R., Dewey M.J., Rong J., Johnson S.A., D’Angelo W.A., Hussey G.S., Badylak S.F. (2024). Matrix-Bound Nanovesicles Alleviate Particulate-Induced Periprosthetic Osteolysis. Sci. Adv..

[18] Mora-Navarro C., Badileanu A., Gracioso Martins A.M., Ozpinar E.W., Gaffney L., Huntress I., Harrell E., Enders J.R., Peng X., Branski R.C. (2020). Porcine Vocal Fold Lamina Propria-Derived Biomaterials Modulate TGF-Β1-Mediated Fibroblast Activation in Vitro. ACS Biomater. Sci. Eng..

[19] Yang J., Bahcecioglu G., Ronan G., Zorlutuna P. (2024). Aged Breast Matrix Bound Vesicles Promote Breast Cancer Invasiveness. Biomaterials.

[20] Chen Z.-H., Hu Y.-R., Yue X.-B., Zhao K., Yang H., Liu Z.-G., Xu R., Lü W.-D. (2025). Matrix-Bound Nanovesicles Isolated from Decellularized Tumors as Platforms for Targeting Parent Tumor Cells and Tumor-Associated Stromal Cells. Mater. Today Bio.

[21] Vit O., Petrak J. (2017). Integral Membrane Proteins in Proteomics. How to Break. Open the Black Box?. J. Proteom..

[22] Sivanantham A., Jin Y. (2022). Impact of Storage Conditions on EV Integrity/Surface Markers and Cargos. Life.

[23] Ahmadian S., Jafari N., Tamadon A., Ghaffarzadeh A., Rahbarghazi R., Mahdipour M. (2024). Different Storage and Freezing Protocols for Extracellular Vesicles: A Systematic Review. Stem Cell Res. Ther..

[24] Anderson H.C. (2003). Matrix Vesicles and Calcification. Curr. Rheumatol. Rep..

[25] Francesco D.D., Mantovani D., Hussey G., Boccafoschi F. (2025). Matrix Bound Nanovesicles: A Great Promise for TERM in Less than a Decade of Research. Matrix Biol..

[26] Ripoll L., Zickler A.M., Vader P., El Andaloussi S., Verweij F.J., van Niel G. (2026). Biology and Therapeutic Potential of Extracellular Vesicle Targeting and Uptake. Nat. Rev. Mol. Cell Biol..

[27] Choi W., Park D.J., Eliceiri B.P. (2024). Defining Tropism and Activity of Natural and Engineered Extracellular Vesicles. Front. Immunol..

[28] Yunusova N.V., Dandarova E.E., Svarovsky D.A., Denisov N.S., Kostromitsky D.N., Patysheva M.R., Cheremisina O.V., Spirina L.V. (2022). Production and Internalization of Extracellular Vesicles in Norm and under Conditions of Hyperglycemia and Insulin Resistance. Biochem. Mosc. Suppl. Ser. B.

[29] Sigismund S., Lanzetti L., Scita G., Di Fiore P.P. (2021). Endocytosis in the Context-Dependent Regulation of Individual and Collective Cell Properties. Nat. Rev. Mol. Cell Biol..

[30] Kumar M.A., Baba S.K., Sadida H.Q., Marzooqi S.A., Jerobin J., Altemani F.H., Algehainy N., Alanazi M.A., Abou-Samra A.-B., Kumar R. (2024). Extracellular Vesicles as Tools and Targets in Therapy for Diseases. Signal Transduct. Target. Ther..

[31] Edelmann M.J., Kima P.E. (2022). Current Understanding of Extracellular Vesicle Homing/Tropism. Zoonoses.

[32] Liu C., Chen X., Ene J., Esmonde C., Kanekiyo T., Zeng C., Sun L., Li Y. (2025). Engineering Extracellular Vesicles Secreted by Human Brain Organoids with Different Regional Identity. ACS Appl. Mater. Interfaces.

[33] Rejman J., Oberle V., Zuhorn I.S., Hoekstra D. (2004). Size-Dependent Internalization of Particles via the Pathways of Clathrin- and Caveolae-Mediated Endocytosis. Biochem. J..

[34] Cieślik M., Bryniarski K., Nazimek K. (2023). Biodelivery of Therapeutic Extracellular Vesicles: Should Mononuclear Phagocytes Always Be Feared?. Front. Cell Dev. Biol..

[35] Faust A., Kandakatla A., van der Merwe Y., Ren T., Huleihel L., Hussey G., Naranjo J.D., Johnson S., Badylak S., Steketee M. (2017). Urinary Bladder Extracellular Matrix Hydrogels and Matrix-Bound Vesicles Differentially Regulate Central Nervous System Neuron Viability and Axon Growth and Branching. J. Biomater. Appl..

[36] Kobayashi M., Negishi J., Ishida N., Hashimoto Y., Sasaki Y., Akiyoshi K., Kimura T., Kishida A. (2024). Effects of the Matrix-Bounded Nanovesicles of High-Hydrostatic Pressure Decellularized Tissues on Neural Regeneration. Sci. Technol. Adv. Mater..

[37] van der Merwe Y., Faust A.E., Sakalli E.T., Westrick C.C., Hussey G., Chan K.C., Conner I.P., Fu V.L.N., Badylak S.F., Steketee M.B. (2019). Matrix-Bound Nanovesicles Prevent Ischemia-Induced Retinal Ganglion Cell Axon Degeneration and Death and Preserve Visual Function. Sci. Rep..

[38] Liu C., Chen X., Liu Y., Sun L., Yu Z., Ren Y., Zeng C., Li Y. (2023). Engineering Extracellular Matrix-Bound Nanovesicles Secreted by Three-Dimensional Human Mesenchymal Stem Cells. Adv. Healthc. Mater..

[39] Tian T., Zhang H.-X., He C.-P., Fan S., Zhu Y.-L., Qi C., Huang N.-P., Xiao Z.-D., Lu Z.-H., Tannous B.A. (2018). Surface Functionalized Exosomes as Targeted Drug Delivery Vehicles for Cerebral Ischemia Therapy. Biomaterials.

[40] Cramer M., Pineda Molina C., Hussey G., Turnquist H.R., Badylak S.F. (2022). Transcriptomic Regulation of Macrophages by Matrix-Bound Nanovesicle-Associated Interleukin-33. Tissue Eng. Part A.

[41] Fernández-Delgado I., Calzada-Fraile D., Sánchez-Madrid F. (2020). Immune Regulation by Dendritic Cell Extracellular Vesicles in Cancer Immunotherapy and Vaccines. Cancers.

[42] Sadtler K., Wolf M.T., Ganguly S., Moad C.A., Chung L., Majumdar S., Housseau F., Pardoll D.M., Elisseeff J.H. (2019). Divergent Immune Responses to Synthetic and Biological Scaffolds. Biomaterials.

[43] Smith M.H., Gao V.R., Periyakoil P.K., Kochen A., DiCarlo E.F., Goodman S.M., Norman T.M., Donlin L.T., Leslie C.S., Rudensky A.Y. (2023). Drivers of Heterogeneity in Synovial Fibroblasts in Rheumatoid Arthritis. Nat. Immunol..

[44] Meng H.-Y., Chen L.-Q., Chen L.-H. (2020). The Inhibition by Human MSCs-Derived miRNA-124a Overexpression Exosomes in the Proliferation and Migration of Rheumatoid Arthritis-Related Fibroblast-like Synoviocyte Cell. BMC Musculoskelet. Disord..

[45] Meng Q., Qiu B. (2020). Exosomal MicroRNA-320a Derived from Mesenchymal Stem Cells Regulates Rheumatoid Arthritis Fibroblast-Like Synoviocyte Activation by Suppressing CXCL9 Expression. Front. Physiol..

[46] Campbell G.P., Amin D., Hsieh K., Hussey G.S., St. Leger A.J., Gross J.M., Badylak S.F., Kuwajima T. (2024). Immunomodulation by the Combination of Statin and Matrix-Bound Nanovesicle Enhances Optic Nerve Regeneration. npj Regen. Med..

[47] Hussey G.S., Dziki J.L., Lee Y.C., Bartolacci J.G., Behun M., Turnquist H.R., Badylak S.F. (2019). Matrix Bound Nanovesicle-Associated IL-33 Activates a pro-Remodeling Macrophage Phenotype via a Non-Canonical, ST2-Independent Pathway. J. Immunol. Regen. Med..

[48] Zhang B., Liu J., Liu L., Wu J., Mo X., Tang R., Liu Z. (2026). Tissue-Specific Matrix-Bound Nanovesicles Regulate the Immunoregulatory Progress of Biological Mesh-Aided Abdominal Hernia Repair. Bioact. Mater..

[49] Tyurina Y.Y., Tyurin V.A., Kapralov A.A., Hussey G.S., Timashev P.S., Shvedova A.A., Badylak S.F., Kagan V.E. (2021). Lipids as Regulators of Inflammation and Tissue Regeneration. Immunomodulatory Biomaterials.

[50] Sadtler K., Allen B.W., Estrellas K., Housseau F., Pardoll D.M., Elisseeff J.H. (2017). The Scaffold Immune Microenvironment: Biomaterial-Mediated Immune Polarization in Traumatic and Nontraumatic Applications. Tissue Eng. Part A.

[51] Laude M., Kolliopoulos V., Mikos A.G., White L.J., Cosgriff-Hernandez E. (2026). Extracellular-Matrix-Based Materials from Decellularized Tissue: Opportunities, Challenges, and Future Directions in Regenerative Medicine. Adv. Healthc. Mater..

[52] Leyendecker P.M., Capella-Monsonís H., Hussey G.S. (2025). Matrix-Bound Nanovesicles: The Extracellular Matrix Vesicle. Regenerative Biomaterials—Emerging Biomaterial Solutions to Aid Tissue Regeneration.

[53] Bartolacci J.G., Behun M.N., Warunek J.P., Li T., Sahu A., Dwyer G.K., Lucas A., Rong J., Ambrosio F., Turnquist H.R. (2024). Matrix-Bound Nanovesicle-Associated IL-33 Supports Functional Recovery after Skeletal Muscle Injury by Initiating a pro-Regenerative Macrophage Phenotypic Transition. npj Regen. Med..

[54] Wang X., Chung L., Hooks J., Maestas D.R., Lebid A., Andorko J.I., Huleihel L., Chin A.F., Wolf M., Remlinger N.T. (2021). Type 2 Immunity Induced by Bladder Extracellular Matrix Enhances Corneal Wound Healing. Sci. Adv..

[55] van der Merwe Y., Faust A.E., Steketee M.B. (2017). Matrix Bound Vesicles and miRNA Cargoes Are Bioactive Factors within Extracellular Matrix Bioscaffolds. Neural Regen. Res..

[56] Baum B.J., Baum G.R., Cox C.T., Valerio I.L., MacKay B.J. (2023). Use and Efficacy of Porcine Urinary Bladder Matrix for Tissue Regeneration: A Review. Wounds.

[57] Valerio I.L., Campbell P., Sabino J., Dearth C.L., Fleming M. (2015). The Use of Urinary Bladder Matrix in the Treatment of Trauma and Combat Casualty Wound Care. Regen. Med..

[58] Brown B.N., Londono R., Tottey S., Zhang L., Kukla K.A., Wolf M.T., Daly K.A., Reing J.E., Badylak S.F. (2012). Macrophage Phenotype as a Predictor of Constructive Remodeling Following the Implantation of Biologically Derived Surgical Mesh Materials. Acta Biomater..

[59] Toth T., Prisca R.-A., Ballo N., Prisca A.-M., Szasz E.A., Borda A. (2025). Porcine Small Intestinal Submucosa Extracellular Matrix: A Meta-Analysis of Composition, Processing Techniques, and Biomedical Applications. Int. J. Mol. Sci..

[60] Mosala Nezhad Z., Poncelet A., de Kerchove L., Gianello P., Fervaille C., El Khoury G. (2016). Small Intestinal Submucosa Extracellular Matrix (CorMatrix^®^) in Cardiovascular Surgery: A Systematic Review. Interact. Cardiovasc. Thorac. Surg..

[61] Liu W., Zhang X., Jiang X., Dai B., Zhang L., Zhu Y. (2023). Decellularized Extracellular Matrix Materials for Treatment of Ischemic Cardiomyopathy. Bioact. Mater..

[62] Fujii M., Tanaka R. (2022). Porcine Small Intestinal Submucosa Alters the Biochemical Properties of Wound Healing: A Narrative Review. Biomedicines.

[63] Wang M., Li D., Ouyang S., Tong B., Chen Y., Ding B., Wang J., Jiang Z., Xu H., Hu S. (2025). Hydrogel Derived from Decellularized Pig Small Intestine Submucosa Boosted the Therapeutic Effect of FGF-20 on TNBS-Induced Colitis in Rats via Restoring Gut Mucosal Integrity. Mater. Today Bio.

[64] Keane T.J., Dziki J., Sobieski E., Smoulder A., Castleton A., Turner N., White L.J., Badylak S.F. (2017). Restoring Mucosal Barrier Function and Modifying Macrophage Phenotype with an Extracellular Matrix Hydrogel: Potential Therapy for Ulcerative Colitis. J. Crohn’s Colitis.

[65] Shariat Rad P., Khazaei M., Ghanbari E., Rashidi M., Rezakhani L. (2025). Recent Advances in Pericardium Extracellular Matrix for Tissue Regeneration, along with a Short Insight into Artificial Intelligence. Front. Med. Technol..

[66] Botes L., Laker L., Dohmen P.M., van den Heever J.J., Jordaan C.J., Lewies A., Smit F.E. (2022). Advantages of Decellularized Bovine Pericardial Scaffolds Compared to Glutaraldehyde Fixed Bovine Pericardial Patches Demonstrated in a 180-Day Implant Ovine Study. Cell Tissue Bank..

[67] Dallatana A., Cremonesi L., Pezzini F., Fontana G., Innamorati G., Giacomello L. (2024). The Placenta as a Source of Human Material for Neuronal Repair. Biomedicines.

[68] Sun J.-Y., Wu R., Xu J., Xue H.-Y., Lu X.-J., Ji J. (2021). Placental Immune Tolerance and Organ Transplantation: Underlying Interconnections and Clinical Implications. Front. Immunol..

[69] Hopkinson A., Figueiredo F.C. (2025). A Narrative Review of Amniotic Membrane Transplantation in Ocular Surface Repair: Unveiling the Immunoregulatory Pathways for Timely Intervention. Ophthalmol. Ther..

[70] Parmar U.P.S., Surico P.L., Scarabosio A., Barone V., Singh R.B., D’Ancona F., Zeppieri M., Parodi P.C., Mori T., Cutrupi F. (2025). Amniotic Membrane Transplantation for Wound Healing, Tissue Regeneration and Immune Modulation. Stem Cell Rev. Rep..

[71] Baig I.F., Le N.T., Al-Mohtaseb Z. (2023). Amniotic Membrane Transplantation: An Updated Clinical Review for the Ophthalmologist. Ann. Eye Sci..

[72] Peshkova M., Korneev A., Revokatova D., Smirnova O., Klyucherev T., Shender V., Arapidi G., Kosheleva N., Timashev P. (2024). Four Sides to the Story: A Proteomic Comparison of Liquid-Phase and Matrix-Bound Extracellular Vesicles in 2D and 3D Cell Cultures. Proteomics.

[73] Tiegs G., Lohse A.W. (2010). Immune Tolerance: What Is Unique about the Liver. J. Autoimmun..

[74] Petrie K., Cox C.T., Becker B.C., MacKay B.J. (2022). Clinical Applications of Acellular Dermal Matrices: A Review. Scars Burn. Heal..

[75] Marguiles I.G., Salzberg C.A. (2019). The Use of Acellular Dermal Matrix in Breast Reconstruction: Evolution of Techniques over 2 Decades. Gland. Surg..

[76] Dussoyer M., Michopoulou A., Rousselle P. (2020). Decellularized Scaffolds for Skin Repair and Regeneration. Appl. Sci..

[77] Kovach J.L., Schwartz S.G., Flynn H.W., Scott I.U. (2012). Anti-VEGF Treatment Strategies for Wet AMD. J. Ophthalmol..

[78] Sassa Y., Hata Y. (2010). Antiangiogenic Drugs in the Management of Ocular Diseases: Focus on Antivascular Endothelial Growth Factor. Clin. Ophthalmol..

[79] Miyaki S., Lotz M.K. (2018). Extracellular Vesicles in Cartilage Homeostasis and Osteoarthritis. Curr. Opin. Rheumatol..

[80] Rowland C.R., Lennon D.P., Caplan A.I., Guilak F. (2013). The Effects of Crosslinking of Scaffolds Engineered from Cartilage ECM on the Chondrogenic Differentiation of MSCs. Biomaterials.

[81] Sutherland A.J., Converse G.L., Hopkins R.A., Detamore M.S. (2015). The Bioactivity of Cartilage Extracellular Matrix in Articular Cartilage Regeneration. Adv. Healthc. Mater..

[82] Abrams G.D., Mall N.A., Fortier L.A., Roller B.L., Cole B.J. (2013). BioCartilage: Background and Operative Technique. Oper. Tech. Sports Med..

[83] Xie J., Hu Y., Li H., Wang Y., Fan X., Lu W., Liao R., Wang H., Cheng Y., Yang Y. (2023). Targeted Therapy for Peri-Prosthetic Osteolysis Using Macrophage Membrane-Encapsulated Human Urine-Derived Stem Cell Extracellular Vesicles. Acta Biomater..

[84] Le Q.-V., Lee J., Lee H., Shim G., Oh Y.-K. (2021). Cell Membrane-Derived Vesicles for Delivery of Therapeutic Agents. Acta Pharm. Sin. B.

[85] Wang A.Y.L., Kao H.-K., Liu Y.-Y., Loh C.Y.Y. (2025). Engineered Extracellular Vesicles Derived from Pluripotent Stem Cells: A Cell-Free Approach to Regenerative Medicine. Burn. Trauma.

[86] Zhang X., Zhang H., Gu J., Zhang J., Shi H., Qian H., Wang D., Xu W., Pan J., Santos H.A. (2021). Engineered Extracellular Vesicles for Cancer Therapy. Adv. Mater..

[87] Anwar I., Wang X., Parlongo S., Harris S., Pratt R.E., Dzau V.J., Hodgkinson C.P. (2026). Engineering Extracellular Vesicles for Targeted Therapeutic Delivery in the Heart. Biosci. Rep..

[88] Ciferri M.C., Bruno S., Rosenwasser N., Gorgun C., Reverberi D., Gagliani M.C., Cortese K., Grange C., Bussolati B., Quarto R. (2024). Standardized Method to Functionalize Plasma-Extracellular Vesicles via Copper-Free Click Chemistry for Targeted Drug Delivery Strategies. ACS Appl. Bio Mater..

[89] Ragab S.S. (2025). Signature of Click Chemistry in Advanced Techniques for Cancer Therapeutics. RSC Adv..

[90] Smyth T., Petrova K., Payton N.M., Persaud I., Redzic J.S., Graner M.W., Smith-Jones P., Anchordoquy T.J. (2014). Surface Functionalization of Exosomes Using Click Chemistry. Bioconjugate Chem..

[91] Zhang S., Chan K.H., Prud’homme R.K., Link A.J. (2012). Synthesis and Evaluation of Clickable Block Copolymers for Targeted Nanoparticle Drug Delivery. Mol. Pharm..

[92] Liu S. (2009). Radiolabeled Cyclic RGD Peptides as Integrin Avβ3-Targeted Radiotracers: Maximizing Binding Affinity via Bivalency. Bioconjugate Chem..

[93] Zhao L., Chen H., Lu L., Zhao C., Malichewe C.V., Wang L., Guo X., Zhang X. (2021). Design and Screening of a Novel Neuropilin-1 Targeted Penetrating Peptide for Anti-Angiogenic Therapy in Glioma. Life Sci..

[94] Ahmad F., Salem-Bekhit M.M., Khan F., Alshehri S., Khan A., Ghoneim M.M., Wu H.-F., Taha E.I., Elbagory I. (2022). Unique Properties of Surface-Functionalized Nanoparticles for Bio-Application: Functionalization Mechanisms and Importance in Application. Nanomaterials.

[95] Kooijmans S.A.A., Fliervoet L.A.L., van der Meel R., Fens M.H.A.M., Heijnen H.F.G., van Bergen en Henegouwen P.M.P., Vader P., Schiffelers R.M. (2016). PEGylated and Targeted Extracellular Vesicles Display Enhanced Cell Specificity and Circulation Time. J. Control. Release.

[96] Choi E.S., Song J., Kang Y.Y., Mok H. (2019). Mannose-Modified Serum Exosomes for the Elevated Uptake to Murine Dendritic Cells and Lymphatic Accumulation. Macromol. Biosci..

[97] Han Y., Jones T.W., Dutta S., Zhu Y., Wang X., Narayanan S.P., Fagan S.C., Zhang D. (2021). Overview and Update on Methods for Cargo Loading into Extracellular Vesicles. Processes.

[98] Mediratta K., Diab M.D., Han P., Hu H., Wang L. (2025). Emerging Strategies for Cargo Loading and Engineering of Extracellular Vesicles for Breast Cancer Treatment. Nanomaterials.

[99] Zeng H., Guo S., Ren X., Wu Z., Liu S., Yao X. (2023). Current Strategies for Exosome Cargo Loading and Targeting Delivery. Cells.

[100] Huang Z., Cheng J., Deng Z., Liu C., Huang T., Lin W. (2025). Extracellular Vesicle-Based Therapeutic Cargo Delivery for Cancer Therapy. Int. J. Nanomed..

[101] Raghav A., Jeong G.-B. (2021). A Systematic Review on the Modifications of Extracellular Vesicles: A Revolutionized Tool of Nano-Biotechnology. J. Nanobiotechnol..

[102] Lamichhane T.N., Raiker R.S., Jay S.M. (2015). Exogenous DNA Loading into Extracellular Vesicles via Electroporation Is Size-Dependent and Enables Limited Gene Delivery. Mol. Pharm..

[103] Singh M., Mazaheri-Tehrani G., Martin-Fabiani I., Davies O.G. (2025). Electroporation Induced Changes in Extracellular Vesicle Profile. Drug Deliv..

[104] Fernández-Rhodes M., Lorca C., Lisa J., Batalla I., Ramos-Miguel A., Gallart-Palau X., Serra A. (2024). New Origins of Yeast, Plant and Bacterial-Derived Extracellular Vesicles to Expand and Advance Compound Delivery. Int. J. Mol. Sci..

[105] Nizamudeen Z.A., Xerri R., Parmenter C., Suain K., Markus R., Chakrabarti L., Sottile V. (2021). Low-Power Sonication Can Alter Extracellular Vesicle Size and Properties. Cells.

[106] Sancho-Albero M., del Mar Encabo-Berzosa M., Beltrán-Visiedo M., Fernández-Messina L., Sebastián V., Sánchez-Madrid F., Arruebo M., Santamaría J., Martín-Duque P. (2019). Efficient Encapsulation of Theranostic Nanoparticles in Cell-Derived Exosomes: Leveraging the Exosomal Biogenesis Pathway to Obtain Hollow Gold Nanoparticle-Hybrids. Nanoscale.

[107] Chen Y., Wang L., Yu X., Mao W., Wan Y. (2024). Ultrasonication Outperforms Electroporation for Extracellular Vesicle Cargo Depletion. Extracell. Vesicle.

[108] Luo L., Avery S.J., Waddington R.J. (2021). Exploring a Chemotactic Role for EVs from Progenitor Cell Populations of Human Exfoliated Deciduous Teeth for Promoting Migration of Naïve BMSCs in Bone Repair Process. Stem Cells Int..

[109] Zhang L., Shi C., Yan L., Zhang X., Ji X., Li L., He X., Tan Y., Zhao N., Lu C. (2025). The Application of Exosomes from Different Sources Loaded with Natural Small-Molecule Compounds in Disease. Int. J. Nanomed..

[110] Huang X., Li A., Xu P., Yu Y., Li S., Hu L., Feng S. (2023). Current and Prospective Strategies for Advancing the Targeted Delivery of CRISPR/Cas System via Extracellular Vesicles. J. Nanobiotechnol..

[111] Piening L.M., Wachs R.A. (2023). Matrix-Bound Nanovesicles: What Are They and What Do They Do?. Cells Tissues Organs.

